# Physiochemical Responses of the Kernel Quality, Total Phenols and Antioxidant Enzymes of Walnut in Different Forms to the Low-Temperature Storage

**DOI:** 10.3390/foods10092027

**Published:** 2021-08-28

**Authors:** Yanping Ma, Chaoye Wang, Chaobin Liu, Jiawei Tan, Huiling Ma, Jin Wang

**Affiliations:** 1 College of Forestry, Northwest A&F University, Xianyang 712100, China; myp1273@163.com (Y.M.); 18220183723@163.com (C.W.); liuchaobin@126.com (C.L.); tanjiawei@nwafu.edu.cn (J.T.); 2 College of Life Science, Northwest A&F University, Xianyang 712100, China; hl65@nwafu.edu.cn; 3 Key Laboratory of Environmental Medicine and Engineering, Ministry of Education, and Department of Nutrition and Food Hygiene, School of Public Health, Southeast University, Nanjing 210009, China; 4 Department of Bioresource Engineering, Faculty of Agricultural and Environmental Sciences, McGill University, Sainte-Anne-de-Bellevue, QC H9X 3V9, Canada

**Keywords:** fresh walnut, total phenols, frozen storage, fatty acid, antioxidant enzyme

## Abstract

Fresh walnut is obtaining high attention due to its pleasant taste and health benefits. This study aimed to evaluate the influence of storage temperatures (0 °C and −20 °C) on the kernel quality, total phenols, and antioxidant enzyme activities of walnuts in three forms (fresh kernels, walnuts with green husk, and walnuts with shell). For a short storage within 3 months at 0 °C, the results revealed that walnuts with green husk provided a better walnut kernel quality resulting from its lower acid value and peroxide value, together with a higher total phenol content and total antioxidant activity, compared with other forms of walnuts. In comparison, frozen storage at −20 °C for a long duration (up to 10 months), found that walnuts with shell showed advantages in improving the kernel quality (fatty acid content, total phenols, and total antioxidant activity) and antioxidant enzyme (peroxidase, catalase, and superoxide dismutase) activities in the kernels, leading to an acceptable range of acid value and peroxide value, compared with other forms of walnuts. Thus, frozen storage at −20 °C showed a potential application in maintaining the walnut kernel quality, especially the walnuts with shell.

## 1. Introduction

Walnut (*Juglans regia* L.) has obtained high attention by producers due to its economic values. In 2017, the Food and Agriculture Organization of the United Nations (FAO) reported that walnut production reached 1,819,400 tons and was valued at 7665 million US dollars in China, accounting for 50% of the total global walnut production (FAOSTAT). Furthermore, walnuts are one of the most popular tree nuts attributing to their nutritional values. The United States Department of Agriculture (USDA) Nutrient Database shows that 100 g of dried walnuts contains 16.67 g of protein, 13.33 g of carbohydrates, 6.7 g of dietary fiber, 440 mg of Potassium, and 97 mg of Calcium [1]. Also, walnuts are rich in health-promoting bioactive compounds, including ω-3 fatty acids, plant sterols, polyphenols, and bioactive peptides [2,3,4]. Many studies have reported that these bioactive compounds can aid against aging, cancers, metabolic syndrome, diabetes, and cardiovascular-related diseases [5,6,7].

Nowadays, consuming walnuts in fresh form is becoming more common due to their superior flavor, higher Vitamin E, polyphenol and antioxidant content compared to walnuts in dried form [8,9,10]. However, fresh walnuts have a very short shelf life due to their high moisture content and perishable characteristics. Previous studies reported that modified atmosphere packaging combined with cold storage can extend the shelf life of fresh walnuts for 2 months [9]. ^60^Coγ-irradiation can improve the shelf life of fresh walnuts up to 3 months [8]. However, the applications of these techniques are limited due to the lack of cold storage room and related equipment in most areas of China. Therefore, more practical methods following less procedures are in need to improve the shelf life of fresh walnuts.

Freezing technology for food preservation has been used by humans for thousands of years. The application of freezing technology has provided many commercial products including broccoli, potatoes, corn, green beans, strawberries, cherries, raspberries, and litchi [11,12,13]. Studies compared these frozen products with fresh ones and found that no significant differences were observed in the color attributes, taste, and nutrient content [14,15]. Further, the freezing processing is easy to undertake in most families with refrigerators/freezers in the modern world.

However, very limited studies were reported regarding the influences of the freezing technology on the quality of fresh walnuts, especially in different forms of fresh walnuts: fresh kernel, fresh walnuts with shell, fresh walnuts with green husk. Therefore, the objective of the present study is to investigate the physiochemical responses of the kernel quality, antioxidant activity, and protective enzymes, including superoxide dismutase (SOD), peroxidase (POD), and catalase (CAT) to low temperatures under three different forms (fresh kernel, fresh walnuts with shell, fresh walnuts with green husk) during freezing. Hopefully, this study can provide technical support for the preservation of fresh walnuts.

## 2. Materials and Methods

### 2.1. Plant Material and Treatments

**Figure 1:**
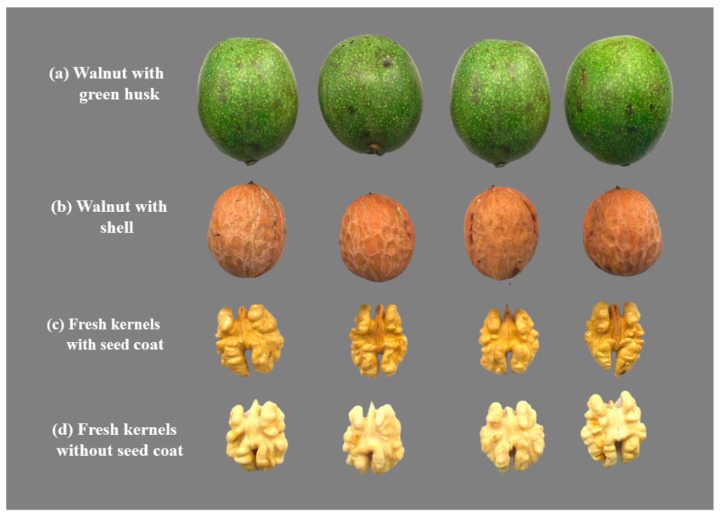
Different forms of fresh walnuts after harvesting: (**a**) walnut with green husk; (**b**) fresh walnut with shell; (**c**) fresh kernels with seed coat; (**d**) fresh kernels without seed coat.

Fresh walnuts (*Juglans regia* L. cv. *Xiangling*) with green husk were harvested from a local farm (Zhouzhi, GPRS: Lo-108.22207; La-34.16337) in Xi’an, Shaanxi Province, China. After harvest, the walnuts were transferred to the lab and pre-cooled at 0 °C for 24 h. Fresh walnuts were prepared in three forms: walnuts with green husk (Figure 1a), walnuts with shell (Figure 1b), and fresh kernels with seed coat (Figure 1c), and were stored at 0 °C and −20 °C. Each treatment was set in triplicate containing 400 walnuts. During the period of storage, nine sample points were set at the months of 0, 1, 2, 3, 4, 5, 6, 8, and 10. Fifteen walnuts were taken at each point, and their kernels were used for related measurements.

### 2.2. Relative Electrical Conductivity (REC)

Walnut kernel powder (3 g) was mixed with 30 mL of double distilled water and was incubated at 25 °C for 1 hr. The electrical conductivity (P1) was determined by using a conductometer (DDS-307A, Inesa-Instrument Co., Ltd., Shanghai, China) [16]. Then, the mixed walnut solution was boiled in a water bath for 15 min. After cooling, the value of the electrical conductivity (P2) was recorded. The electrical conductivity of the walnut samples was calculated using the following equation:

(1) $$REC(\%)=\frac{\left(P_{1}-P_{0}\right)}{\left(P_{2}-P_{0}\right)}\times 100\%$$

### 2.3. Total Phenol Analysis

According to the method described by Wang et al. (2019), one gram of the walnut sample was mixed with 10 mL of 70% ethanol (*v*/*v*), and the mixture was extracted at room temperature for 30 min. After centrifuging at 8000× *g* for 10 min, the supernatant was stored at 4 °C for the analyses of total phenols and antioxidant activity. The total phenol content of the walnut kernel was determined using the Folin-Ciocalteau assay [17]. Specifically, 0.2 mL of extracts were mixed with 6 mL of double-distilled water, 0.5 mL of Folin-Ciocalteau reagent, and 1.5 mL of a 20% sodium carbonate solution. The mixture was transferred at 75 °C for 10 min, and then the absorbance changes were recorded at 765 nm using a spectrophotometer (UV-3100, Mapada Co. Ltd., Shanghai, China). Gallic acid was used to obtain the standard curve.

### 2.4. Antioxidant Activity

#### 2.4.1. Ferric Reducing Antioxidant Power (FRAP) Assay

According to the method mentioned by Benzie and Strain (1996), an FRAP working solution was obtained by mixing together an acetic acid buffer (40 mM), a ferric chloride solution (20 mM), and 2,4,6-Tripyridyl-S-triazine (TPTZ) by the rate of 1:1:10 (*v*/*v*/*v*) [18]. The walnut kernel extract (0.2 mL) was incubated with 3 mL of FRAP at 37 °C for 30 min. Then, the absorbance of the mixture was recorded at 593 nm. The standard curve was obtained using a 1.0 mol/L FeSO_4_ solution, and the results were expressed as mmol FeSO_4_/g FW of walnut kernels.

#### 2.4.2. DPPH Radical Scavenging Activity

DPPH radical scavenging activity was another method used to evaluate the antioxidant activity of the walnut kernels. The walnut kernel extract was incubated with a DPPH solution by the rate of 1:1 at room temperature for 30 min. The color changes were recorded at 517 nm. According to the method described by Wang et al. (2019), the antioxidant activity was calculated by using the following equation:

(2) $$DPPH(\%)=\frac{\left(Abs_{0}-Abs_{1}\right)}{Abs_{0}}\times 100$$

where *Abs_0_* is the absorbance value of the blank sample, and *Abs_1_* is the absorbance of samples [17].

### 2.5. Activities of Antioxidant Enzymes: CAT, SOD, and POD

One gram of the walnut kernel sample was grounded with 8 mL of a pre-cooled phosphate buffer (0.1 M, pH 6.8) to extract the relevant antioxidant enzymes [10]. After 30 min incubation at 4 °C, the mixture was centrifuged at 10,000× *g* for 10 min. The supernatant was collected and stored at 4 °C until further analysis.

The CAT activity of the walnut kernels was measured by mixing 400 μL of an enzyme extract, 6 mL of the phosphate buffer (0.1 M, pH 6.8), and 0.2 mL of H_2_O_2_ (2%). One unit of CAT activity was defined as an increase of 0.01 absorbance unit per min at 240 nm [19]. The activity of SOD was obtained using 3 mL of a working solution containing 100 μL of enzyme extract and nitroblue tetrazolium. One unit of SOD activity is defined as the amount of enzyme that causes 50% inhibition of nitroblue tetrazolium [19]. The absorbance changes of a mixed solution containing 1 mL of enzyme extract, 1 mL of phosphate buffer (0.1 M, pH 6.8), 3 mL of guaiacol (25 mM), and 0.2 mL of H_2_O_2_ (2%) was recorded at 410 nm for 5 min to analyze the POD activities of the walnut kernels. One unit of POD activity was defined as an increase of 0.01 absorbance units per min [9].

### 2.6. Changes of O_2_^−^, H_2_O_2_, and MDA Contents in Walnut Kernels

The walnut kernels (5 g) were grounded with 15 mL of a pre-cooled phosphate buffer (0.01 M, pH 7.0) and homogenized at 4 °C for 45 min [20]. After centrifuging at 10,000× *g* for 10 min, the supernatant was collected for the analyses of O_2_^−^ and H_2_O_2_ production. The generation of O_2_^−^ was measured by adding 0.5 mL of the supernatant, 0.5 mL of the pre-cooled phosphate buffer (0.01 M, pH 7.0), and 0.2 mL of hydroxylamine hydrochloride (0.01 M). After incubation at 25 °C for 1 h, 1 mL of p-aminobenzenesulfonic acid (17 mM) and 1 mL of α-naphthyl amine (0.05 M) was added. The color absorbance was measured at 530 nm. Standard curving was obtained using sodium nitrite to calculate the generation of O_2_^−^ and the results were expressed as mmol/g FW [20].

For the determination of the H_2_O_2_ content, 0.4 mL of the supernatant was mixed with 0.4 mL of the phosphate buffer (0.01 M, pH 7.0), and 0.8 mL of a working solution (potassium dichromate: glacial acetic acid: water = 1:5:15, *w*/*v*/*v*) [9]. After incubating at room temperature for 15 min, the color changes were recorded at 570 nm, and the content of H_2_O_2_ was expressed as mmol/g. Frozen walnut samples were extracted with 10% of a trichloroacetic acid buffer and incubated at room temperature for 30 min [21]. After centrifuging at 8000× *g* for 20 min, 0.5 mL of the supernatant was mixed with 0.5 mL of thiobarbituric acid (0.5%) and incubated at 100 °C for 15 min, and then the mixture was determined at 450 nm, 532 nm, and 600 nm, respectively. The concentration of MDA was expressed as µmol/g.

### 2.7. Acid Value (AV) and Peroxide Value (PV)

In the study, 8 g of fresh walnut kernels were dried at 85 °C. Petroleum ether (boiling range, 30–60 °C) was used as a solvent to extract the oil sample at 40 °C for 12 h through a Soxhlet extractor. The oil sample was stored at 4 °C for the analyses of the acid value, peroxide value and fatty acids composition. The standard of GB/T5009.229-2016a and GB/T 5009.229-2016b was used to evaluate the acid value and peroxide value of the walnut kernels, respectively [22,23]. Each analysis was performed in triplicate.

### 2.8. Fatty Acids Composition

A gas chromatography (6890N, Agilent Technologies, Wokingham, UK) equipped with a DB5 column (0.32 mm × 30 cm) and a flame ionization detector (FID) were used to determine the fatty acid composition in the walnut kernels, according to the method as described by Esteki et al. (2017), with some modifications. The flow rate of the nitrogen gas and H_2_ gas was set at 40 mL/min [24]. The injection volume of the oil sample was 0.3 μL, with a split ratio at 30:1. The initial oven temperature was set at 150 °C and held for 3 min. The oven temperature was set to increase to 220 °C at the rate of 5 °C/min and held for 10 min. The injector and detector temperatures were set at 260 °C [24]. Polyunsaturated fatty acid (PUFA) and unsaturated fatty acid (UFA) contents were calculated according to the contents of individual fatty acids. The ChemStation software was used to analyze data obtained from the study.

### 2.9. Statistical Analysis

All the treatments and analyses were performed in triplicate. Results obtained from the experiment were compared by a one-way analysis of variance (ANOVA) using SPSS 22.0 software (SPSS Inc., Chicago, Illinois, USA). All the data was represented by mean values ± standard deviation (SD), and the significant differences of mean values between samples were evaluated using Duncan’s multiple range test (*p* ≤ 0.05).

## 3. Results and Discussion

### 3.1. Relative Electrical Conductivity (REC)

**Figure 2:**
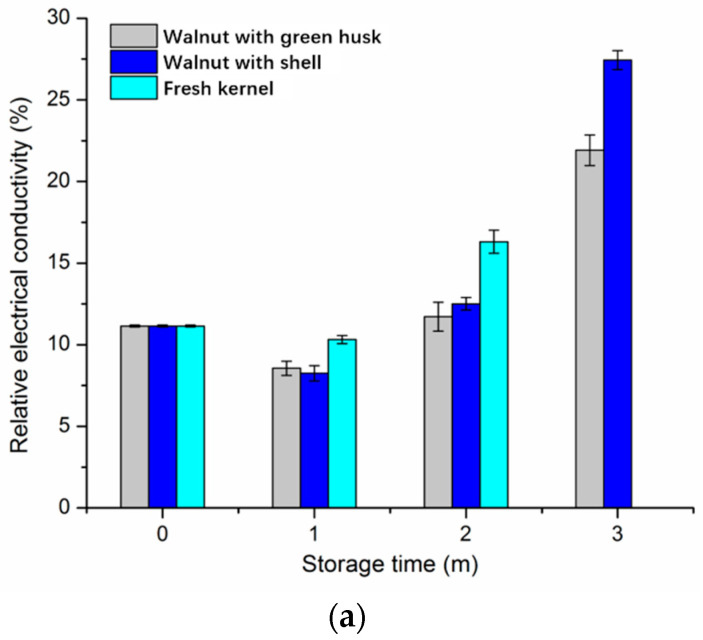

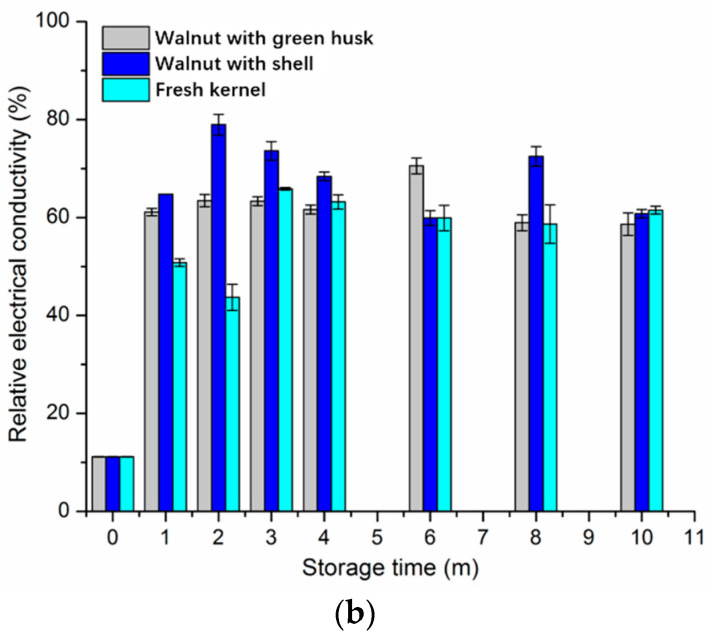
Relative electrical conductivity of walnut kernels under different storage conditions: (**a**) 0 °C and (**b**) −20 °C. Note: m means month in the figure. Data of fresh kernel stored at 0 °C are not available due to the limited shelf life.

As shown in Figure 2, an increasing trend in the REC of walnut kernels was observed under different storage conditions. Specifically, no significant difference in the REC was detected in kernels obtained from walnuts with shell and walnuts with green husk on the 2-month storage at 0 °C, while the fresh kernels showed a higher REC. In comparison, a two-fold increase in the REC of kernels present in the walnuts with green husk and walnuts with shell was observed by the end of a 3-month storage of (Figure 2a). The REC was considered as an indicator to evaluate the kernel quality of nuts through testing the concentration of leachates in the kernel soaking solution, reflecting the damage degree and integrity of the cell membrane [25]. The walnuts with shell and green husk showed a lower conductivity compared with fresh kernels, which indicates shell and green husk can prevent kernels from cell membrane damage contributing to a high physiological potential, during the first 2-month storage at 0 °C. Thereafter, the significant increase in the REC might be due to the very limited shelf life when stored at 0 °C [10].

In comparison, a six-fold increase in the REC of kernels was observed by the end of the 1-month storage at −20 °C, when compared with the initial level (Figure 2b). Then, for the next 10-month storage, the REC of kernels was maintained at a similar level. It indicates that frozen storage remarkably accelerates the damage to the cell membranes of walnut kernels in all three forms, which is primarily attributed to the growth of ice crystals in the cells, resulting in the breakage of kernel cells [26].

### 3.2. Total Phenols Content

As shown in Table 1, the total phenol content of the walnut kernels increased during the 3-month storage at 0 °C. The highest phenol content was observed in the walnut kernels with green husk (72.82 mg/100 g FW), followed by the walnuts with shell (65.44 mg/100 g FW) on the third month, while the values of fresh kernels were not available due to the limited shelf life at 0 °C. In comparison, the total phenol content of the walnut kernels stored at −20 °C showed a fluctuating trend. The total phenol content of the fresh kernels increased up to 85.36 mg/100 g from the initial level (44.23 mg/100 g FW) in the second month, and then decreased to 30.55 mg/100 g FW at the end of storage (Table 1). For the walnuts with shell, the total phenols showed a significant enhancement from 44.23 to 77.43 mg/100 g FW after a 1-month storage, and then it was maintained at a stable range of 51.37–75.89 mg/100 g FW during the subsequent duration. While the total phenol content was maintained at a stable level (44.23–53.84 mg/100 g FW) in the walnuts with green husk during the first 6-month storage at −20 °C, it then showed a two-fold increase to 115.98 mg/100 g FW by the end of the 10-month storage.

A higher level of total phenols was observed in the walnuts with shell and green husk compared with the fresh kernels during the storage. After the 10-month storage at −20 °C, the total phenols present in the walnuts with green husk was increased by 2–4 times higher when compared with the initial level. Similarly, in hazelnuts, a study found that a low temperature (−25 °C) can maintain the phenol concentration of kernels during a 12-month storage [27]. This increase in the total phenol content might be strongly associated with the presence of shell and green husk, which can prevent the phenols from oxidation. Studies have reported that a transformation of compounds between the green husk and kernels of walnuts continues after being picked from the trees, which in turn could contribute to the synthesis of phenols in the kernels during storage [9,10]. The dramatic increase in the total phenols of walnuts with green husk in the late storage stage (8–10 months) might be attributed to the closed environmental stresses obtained from the green husk under the frozen conditions.

### 3.3. Total Antioxidant Activity (TAC)

In the present study, two methods, including FRAP and DPPH assays, were used to quantify the antioxidant activity of the walnut kernels. As shown in Table 1, the results found that the TAC of kernels was improved when stored at 0 °C using FRAP assay. The highest antioxidant activity was observed in the walnuts with green husk (4.90 mmol/g), followed by the walnuts with shell (4.71 mmol/g), while the data of fresh kernels was not available because of their short shelf life. It indicates that walnuts, especially the form with green husk, showed advantages in maintaining or improving the TAC of kernels during a short period of storage at 0 °C. However, no significant difference in the TAC of walnut kernels in three forms was observed when stored at −20 °C. During the 10-month storage, the highest average value of TAC was observed in the walnuts with shell (3.57 mmol/g), followed by fresh kernel (3.39 mmol/g), and walnuts with green husk (2.79 mmol/g). The higher presence of TAC in the walnuts with shell was strongly associated with its higher total phenol content (Table 1). In addition, the results revealed that frozen storage could be beneficial to maintaining the TAC of walnut kernels.

In regards to the DPPH radical scavenging activity, similar results were observed during the storage at 0 °C (Table 1). The DPPH radical scavenging activity in the fresh kernels and walnuts with shell significantly increased to 21% from the initial level of 16%, while a decrease in the walnuts with husk (14%) was detected by the end of the 2-month storage at 0 °C. During the 10-month frozen storage at −20 °C, the DPPH radical scavenging activity of walnuts with shell gradually increased from 16% to 26%. However, a dramatic decrease (10–11%) in the DPPH radical scavenging activity was investigated in the fresh kernel and walnuts with green husk at the end of the 2-month storage. This fluctuating in the DPPH radical scavenging activity might be attributed to the total phenol content and TAC. Similarly, many studies have reported that a positive correlation between DPPH and total phenols was observed in mangoes, apples, and tomato fruit [28,29,30].

### 3.4. SOD, CAT, and POD Activity

**Figure 3:**
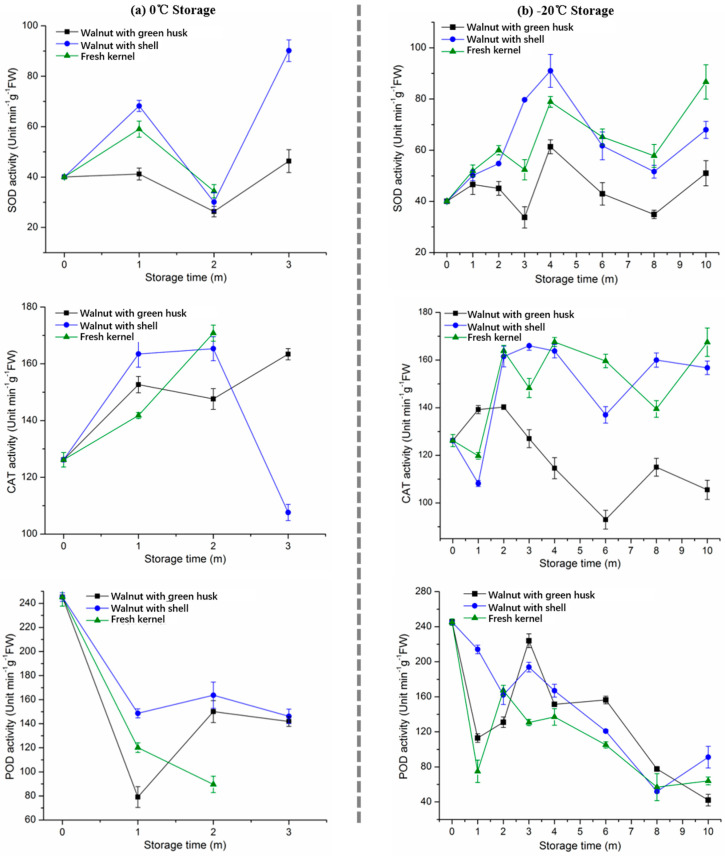
SOD, CAT, and POD activity of walnut kernel under different storage conditions: (**a**) 0 °C and (**b**) −20 °C. Note: m means month in the figure.

SOD, CAT, and POD are three key enzymes which play important roles in catalyzing superoxide radicals, hydrogen peroxides and hydroperoxides into harmless molecules (H_2_O_2_/alcohol and O_2_) [31]. In the present study, these three enzymes showed a fluctuating trend when stored at different conditions (Figure 3). During the first month storage at 0 °C, the SOD activity of the kernels was enhanced and decreased to the initial level in the next month storage, and then a jump enhancement was observed at the end of the 3-month storage (Figure 3a). When frozen storage at −20 °C was applied, walnuts with green husk showed a stable level of SOD with a small peak at the end of the 4-month storage, whereas the peak value of SOD in the walnuts with shell and green husk increased by 2–3 folds compared with the initial level (Figure 3b). After a 6-month storage, the SOD activity of the kernels was maintained at a stable level. A higher level of SOD was observed in the frozen stored walnut samples, which may be due to more SOD being generated in the kernel against low-temperature stress [32,33].

In comparison, an increasing trend in the CAT activity was detected in the kernels when stored at 0 °C during the first 2-month storage, and then a dramatic decrease was observed in the walnuts with shell (Figure 3a). During the storage at −20 °C, the CAT activity of the kernels obtained from the walnuts with shell and green husk significantly increased by 35% from the initial level at the end of the 2-month storage, and then maintained at a stable range (140–160 U min/g FW). In comparison, the CAT activity of the walnuts with green husk started to decrease to a stable range (80–100 U min/g FW) after a 2-month frozen storage at −20 °C. It suggests that green husk played a role in inhibiting the activity of CAT when stored at −20 °C for 10-months, which might be related to the closed environmental condition generated by the green husk. In regards to the POD activity, the results presented a significant decrease (85%) compared with the initial level during the storage at 0 °C and −20 °C (Figure 3a,b). It indicates that low-temperature storage contributed to inhibiting the activity of the POD present in the kernel during the storage.

### 3.5. MDA, H_2_O_2_, and O_2_^−^ Production

**Figure 4:**
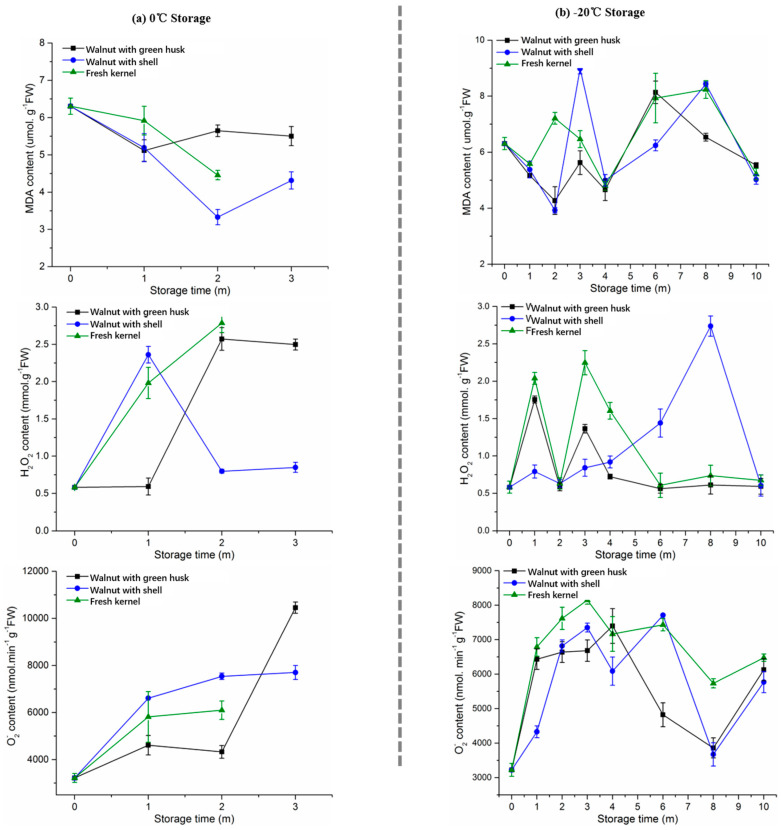
MDA, H_2_O_2_, and O_2_^−^ production of walnut kernel under different storage conditions: (**a**) 0 °C and (**b**) −20 °C. Note: m means month in the figure.

MDA was considered as an important marker to evaluate the membrane lipid peroxidation during the storage of fruits and nuts [20,34]. As shown in Figure 4, the production of MDA in walnut kernels stored at 0 °C decreased with the rise of storage time, while an increasing trend was observed when stored at −20 °C, especially after the 2-month storage. The highest average value of MDA production was detected in the fresh walnut kernels (6.47 μmol/g FW), followed by the walnuts with shell (6.16 μmol/g FW) and walnuts with green husk (5.78 μmol/g FW) (Figure 4b). The results indicate that green husk could contribute to a lower MDA content in the walnut kernels when stored at −20 °C compared with the other forms of walnuts.

In comparison, the production of H_2_O_2_ in the kernels under various conditions showed an increasing trend with the increase of storage time (Figure 3a,b). During the first month of storage at 0 °C, the production of H_2_O_2_ in the fresh kernels and the kernels obtained from walnuts with shell increased by four-fold compared with the initial level, while the kernels obtained from the walnuts with the green husk maintained a stable level of H_2_O_2_. During the frozen storage at −20 °C, the production of H_2_O_2_ in the fresh kernels and walnuts with shell increased sharply during the first 4-month storage and then maintained at the initial level. However, the walnuts with shell delayed the presence of the peak of H_2_O_2_ maintaining within a stable range (0.6–0.8 mmol/g FW), and then the H_2_O_2_ was synthesized abundantly from the 4-month to the 8-month storage at −20 °C. Thus, the inhibiting effect on the production of H_2_O_2_ obtained from the low temperatures was different in various storage conditions. Similarly, an increasing trend was observed in the production of O_2_^−^ in the kernels during the first 3-month storage at 0 °C and −20 °C. After the 4-month frozen storage, the production of O_2_^−^ was reduced to a stable level. Thus, frozen storage can inhibit the production of O_2_^−^ in the kernels of late storage duration.

### 3.6. Fatty Acid Composition

**Figure 5:**
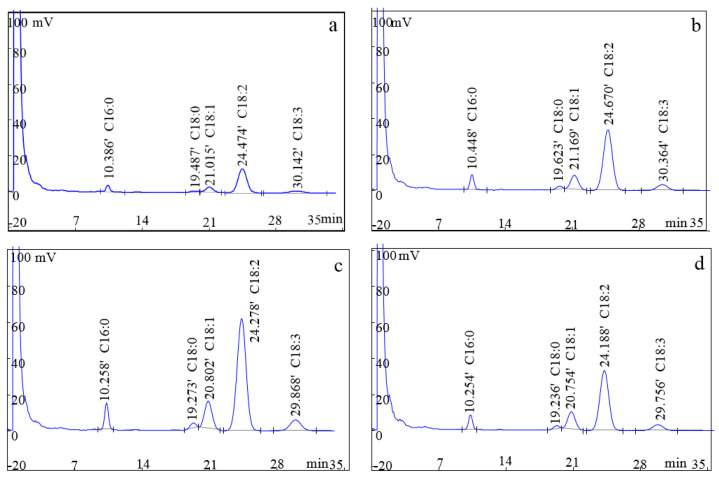
Fatty acid profiles of walnut kernels: (**a**) prior to frozen storage; (**b**) fresh kernel after 8-m frozen storage; (**c**) walnut with shell after 8-m frozen storage; (**d**) walnut with green husk after 8-m frozen storage.

Fatty acids present in the nuts are considered as the key indicator for evaluating the kernel quality [8]. As shown in Table 2, the changes of fatty acid in the kernels stored at −20 °C were observed, while the relevant data was not available because of the limited shelf life of fresh kernels when stored at 0 °C. Five compositions of fatty acids, including palmitic acid, stearic acid, oleic acid, linoleic acid, and α-linolenic acid, were detected using gas chromatography. Among them, the palmitic acid (16:0) content in three forms of walnut kernels decreased from the initial level (9.21%) to 7.29–7.76% after an 8-month storage at −20 °C. Whereas, the oleic acid (18:0) and unsaturated fatty acid level significantly increased in the kernels obtained from the three forms of walnuts (fresh kernel, walnuts with shell, and walnuts with green husk) compared with their initial concentration. In addition, no significant difference in the stearic acid (18:1), linoleic acid (18:2), linolenic acid (18:3), and polyunsaturated fat content of the kernels was observed during the 8-month storage at −20 °C. As shown in Figure 5, the highest peak area of five compositions of fatty acids was observed in the walnuts with shell, followed by fresh kernels and walnuts with green husk at the end of the 8-month storage at −20 °C. The results indicate that frozen storage maintained the majority of fatty acid content, especially in the kernels of walnuts with shell. In *Canarium* nuts, a significant reduction in the fatty acid of frozen kernels compared with the fresh kernels [35]. In butter, a study showed no significant changes in the fatty acid content during a 24-month frozen storage at −20 °C [36]. Thus, the property changes in fatty acids are different in various food samples.

### 3.7. Total Fat Content, Acid Value, and Peroxide Value of Walnut Kernels

As shown in Table 3, a 20% decrease of fat content in the kernels was observed from walnuts with shell and walnuts with green husk stored at 0 °C, compared with the initial value, whereas only a slight reduction in the fat content was detected during the frozen storage at −20 °C. Similar results have been reported by Ma et al. (2013), where they found walnuts stored at 0 °C still perform energy metabolism, resulting in a degradation of fat in the kernels, while frozen storage decreased this effect [8]. Among the three forms of walnuts, no significant difference in the fat content was observed during the 8-month frozen storage at −20 °C. It indicates that frozen storage could contribute to delaying the degradation of fat present in the kernels. In addition, the high maintenance of fat content may be attributed to the high concentration of fatty acid during storage (Table 2).

The acid value of the kernels was used to evaluate the degree of the degradation in the fatty acids during storage, and the higher acid value contributes to a higher free fatty acid content resulting in a lower fat quality of walnut kernels [37]. As shown in Table 3, the results found a slight increase in the acid value of the walnut kernels after a 3-month storage at 0 °C. The walnuts with green husk (0.67 mg/g) showed a lower acid value compared to the walnuts with shell (0.76 mg/g), which might be strongly associated with the higher total phenol content and total antioxidant activity. During the 8-month storage at −20 °C, the acid value of the walnut kernels increased gradually, and the highest acid value was observed in the walnuts with shell (0.84 mg/g), followed by walnuts with green husk (0.68 mg/g) and fresh kernel (0.61 mg/g). No significant difference was observed between the three forms of walnuts at the end of the 8-month frozen storage at −20 °C. It indicates that low-temperature storage showed a potential advantage in preventing fatty acids from oxidation and degradation.

In regards to the peroxide value of the walnut kernels, no significant difference was detected at the end of a 3-month storage at 0 °C when compared with the initial level (Table 3). However, the peroxide value of the kernels increased up to 0.84 mmol/kg from the initial level of 0.59 mmol/kg when stored at −20 °C, and then it decreased to a similar value with the initial level. The results indicate that long-term frozen storage (up to 8 months) at −20°C can contribute to maintaining the peroxide value of walnut kernels. Many studies have reported that phenolic compounds can contribute to the lower acid value, peroxide, which in turn could improve the kernel quality of nuts during storage. In fresh walnuts, Wang et al. (2016) and Ma et al. (2020) observed that phenols played a primary role in reducing the lipid oxidation in the kernels, resulting in a negative correlation between the phenol content and the concentration of acid value and peroxide value [10,38]. In hazelnuts, a study found that frozen storage at −25 °C maintained the kernel quality as a result of the high phenolic composition and antioxidant capacity of kernels during the storage [27]. Similarly, in this study, our results observed a higher total phenol content and DPPH in the walnuts during the storage at −20 °C compared with the initial level, resulting in acceptable acid and peroxide values of the kernels (Table 3).

## 4. Conclusions

In this study, three forms of walnuts, including walnuts with green husk, walnuts with shell, and fresh kernels, were stored at 0 °C and −20 °C. The results found that walnuts with green husk showed a better kernel quality resulted from a lower acid value and peroxide value, and a higher antioxidant activity compared with other forms of walnuts, when stored at 0 °C for a short duration (3 months). In contrast, during the frozen storage at −20 °C for a long duration (up to 10 months), the findings revealed that walnuts with shell showed advantages in improving the fatty acid content, total phenols, and total antioxidant activity compared with other forms of walnuts. Further, the production of H_2_O_2_ and O_2_^−^ in the kernels was inhibited or delayed because of the higher SOD, CAT, and POD activities, which in turn led to maintaining the acid value (AV) and peroxide value (PV) in an acceptable range. In addition, the walnuts with shell saved space for the storage compared with the walnuts with green husk. Therefore, the walnuts with shell showed a potential to be used for the future frozen storage at −20 °C for long-term storage (up to 10 months) in the food industry. However, the relevant physiochemical mechanisms under the cold storage of fresh walnuts are still not clear, and relevant studies are needed in future research.

## Figures and Tables

**Table 1:** Total phenols content and antioxidant activity of walnut kernels during the storage of 0 °C (a) and −20 °C (b). Note: Na means not available. Values with different lowercase letters in the same column ‘a–d’ and uppercase letter ‘A–C’ in the same row are significantly different (*p* < 0.05) from each other during storage.

Storage Time (Month)		Total Phenols (mg/100 g FW)			FRAP (mmol/g FW)			DPPH (%)		
		Fresh Kernel	Walnut with Shell	Walnut with Husk	Walnut Kernel	Walnut with Shell	Walnut with Husk	Fresh Kernel	Walnut with Shell	Walnut with Husk
**0 °C**	0	44.23 ± 0.62Ab	44.23 ± 0.62Ac	44.23 ± 0.62Ac	2.92 ± 0.26Ab	2.92 ± 0.26Ac	2.92 ± 0.26Ab	16.09 ± 1.25Ab	16.09 ±1.25 Ab	16.09 ± 1.25Ab
	1	48.55 ± 0.55Ba	35.77 ± 1.02Cd	59.62 ± 0.73Ab	5.67 ± 0.24Aa	4.31 ± 0.16Aa	4.16 ± 0.22Aa	16.68 ± 0.85Bb	17.48 ± 1.13Bb	22.42 ± 0.45Aa
	2	46.24 ± 1.32ab	51.35 ± 1.51b	57.52 ± 0.94Abc	4.06 ± 0.33Aab	3.66 ± 0.10Aab	2.92 ± 0.13Ab	21.31 ± 1.14Aa	21.67 ± 1.24Aa	14.30 ± 1.83Bb
	3	Na	65.44 ± 2.11Ba	72.82 ± 0.88Aa	Na	4.71 ± 0.11Aa	4.90 ± 0.15Aa	Na	20.94 ± 0.70Aa	23.48 ± 0.77Aa
**−20 °C**	1	55.00 ± 1.51Bb	77.43 ± 1.77Aa	53.00 ± 0.88Bc	3.94±0.41Aa	3.97 ± 0.29Aa	2.57±0.35Ab	21.29 ± 0.79Aab	16.27 ± 0.75Ac	18.46 ± 1.72Ab
	2	85.36 ± 1.85Aa	55.98 ± 1.56Bc	53.84 ± 1.26Bc	3.73 ± 0.23Aa	2.94 ± 0.28Ab	3.21 ± 0.10Aa	11.44 ± 1.19Bc	21.04 ± 0.19Ab	10.83 ± 0.69Bc
	3	56.99 ± 1.57Bb	72.85 ± 2.02Aa	49.89 ± 2.05Bc	3.62 ± 0.38Aa	4.15 ± 0.17Aa	3.16 ± 0.15Aa	23.69 ± 0.44Aa	26.39 ± 0.50Aa	21.66 ± 0.24Ab
	6	47.35 ± 1.47Cc	66.29 ± 1.73Ab	52.44 ± 1.54Bc	3.68 ± 0.46Aa	3.84 ± 0.29Aab	2.27 ± 0.29Ab	24.62 ± 0.46Aa	21.26 ± 0.06Ab	18.09 ± 0.36Ab
	8	47.48 ± 2.25Cc	75.89 ± 2.65Ba	96.10 ± 2.10Aa	2.99 ± 0.46Aab	3.19 ± 0.46Ab	3.12 ± 0.13Aa	16.68 ± 0.29Ab	20.72 ± 0.35Ab	19.96 ± 0.18Ab
	10	30.55 ± 1.31Cd	51.37 ± 1.31Bc	115.98 ± 0.77Aa	2.41 ± 0.25Ab	3.34 ± 0.48Ab	2.44 ± 0.17Ab	24.13 ± 1.49Aa	23.38 ± 0.41Aab	26.78 ± 1.30Aa

**Table 2:** Fatty acid changes of walnut kernels during the storage of −20 °C. Note: Values with different lowercase letters in the same column ‘a–c’ and uppercase letter ‘A–B’ in the same row are significantly different (*p* < 0.05) from each other during storage.

Fatty Acid	Storage Time (Month)	Fatty Acid Composition (%)		
		Fresh Kernel	Walnut with Shell	Walnut with Green Husk
Palmitic acid (16:0)	0	9.21 ± 0.01Aa	9.21 ± 0.01Aa	9.21 ± 0.01Aa
	3	7.86 ± 0.01Ab	8.62 ± 0.06Aab	7.46 ± 0.62Ab
	8	7.29 ± 0.78Ab	7.76 ± 0.85Ac	7.30 ± 0.78Ab
Stearic acid (18:0)	0	2.42 ± 0.16Aa	2.42 ± 0.16Aa	2.42 ± 0.16Aa
	3	2.52 ± 0.01Aa	2.54 ± 0.14Aa	2.41 ± 0.04Aa
	8	2.08 ± 0.01Aa	2.03 ± 0.11Aa	2.18 ± 0.36Aa
Oleic acid (18:1)	0	13.04 ± 0.38Aa	13.04 ± 0.38Aa	13.04 ± 0.38Aa
	3	12.69 ± 0.05Aa	11.18 ± 0.26Aa	13.10 ± 0.48Aa
	8	13.44 ± 0.62Aa	14.54 ± 0.28Aa	13.58 ± 0.62Aa
Linoleic acid (18:2)	0	66.90 ± 0.39Aa	66.90 ± 0.39Aa	66.90 ± 0.39Aa
	3	68.20 ± 0.11Aa	67.93 ± 0.08Aa	68.32 ± 0.11Aa
	8	68.74 ± 0.03Aa	67.35 ± 0.93Aa	67.86 ± 0.45Aa
Linolenic acid (18:3)	0	8.44 ± 0.15Aa	8.44 ± 0.15Aa	8.44 ± 0.15Aa
	3	8.74 ± 0.17Aa	9.73 ± 0.14Aa	8.73 ± 0.31Aa
	8	8.45 ± 0.01Aa	8.32 ± 0.26Aa	9.09 ± 0.01Aa
Polyunsaturated fat (PUFA)	0	75.33 ± 0.92Aa	75.33 ± 0.92Aa	75.33 ± 0.92Aa
	3	76.93 ± 0.48Aa	77.66 ± 0.69Aa	77.05 ± 0.18Aa
	8	77.19 ± 0.38Aa	75.66 ± 0.41Aa	76.95 ± 0.35Aa
Unsaturated fatty acid (UFA)	0	88.37 ± 0.92Aa	88.37 ± 0.92Aa	88.37 ± 0.92Aa
	3	89.62 ± 0.33Aa	88.83 ± 0.43Aa	90.14 ± 1.43Aa
	8	90.63 ± 0.69Aa	90.20 ± 1.66Aa	90.52 ± 1.75Aa

**Table 3:** Total fat content, acid value and peroxide value of walnut kernels during the storage at 0 °C and −20 °C. Note: Na means not available. Values with different lowercase letters in the same column ‘a–b’ and uppercase letter ‘A–B’ in the same row are significantly different (*p* < 0.05) from each other during storage.

Parameters	Storage Time (Month)	0 °C			−20 °C		
		Fresh Kernel	Walnut with Shell	Walnut with Green Husk	Fresh Kernel	Walnut with Shell	Walnut with Green Husk
**Fat content** **(%)**	0	54.04 ± 2.05A	54.04 ± 2.05Aa	54.04 ± 2.05Aa	54.04 ± 2.05Aab	54.04 ± 2.05Aa	54.04 ± 2.05Aa
	3	Na	44.16± 2.42Ab	44.22± 1.93Ab	56.29 ± 0.86Aa	48.29 ± 2.98Bb	50.84 ± 1.05Ba
	8				50.48 ± 3.66Ab	49.50 ± 0.93Ab	51.33 ± 4.10Aa
**Acid value** **(mg/g)**	0	0.57 ± 0.11A	0.57 ± 0.11Ab	0.57 ±0.11Aa	0.57 ± 0.11Aa	0.57 ± 0.11Ab	0.57 ± 0.11Aa
	3	Na	0.76 ± 0.16Aa	0.67 ± 0.03Aa	0.59 ± 0.01Aa	0.67 ± 0.20Ab	0.60 ± 0.05Aa
	8				0.61 ± 0.09Ba	0.84 ± 0.08Aab	0.68 ± 0.02ABa
**Peroxide value** **(mmol/kg)**	0	0.59 ± 0.06A	0.59 ± 0.06Aa	0.59 ± 0.06Aa	0.59 ± 0.06Aab	0.59 ± 0.06Ab	0.59 ± 0.06Aab
	3	Na	0.55 ± 0.00Aa	0.49 ± 0.01Aa	0.79 ± 0.14Aa	0.68 ± 0.09Aab	0.84 ± 0.14Aa
	8				0.37 ± 0.06Bb	0.75 ± 0.10Aa	0.44 ± 0.07Bb

## Data Availability

Not applicable.
